# Psychedelic‐assisted therapy for functional neurological disorders: a review of the literature

**DOI:** 10.1192/j.eurpsy.2025.592

**Published:** 2025-08-26

**Authors:** T. F. Fernandes, J. G. Freire, M. C. Pais, C. Pissarra

**Affiliations:** 1 Centro de Responsabilidade Integrada de Psiquiatria, Unidade Local de Saúde de Coimbra, Coimbra, Portugal

## Abstract

**Introduction:**

Functional neurological disorders (FND), also referred to as conversion disorder or psychogenic neurological disorders, are one of the most common and disabling conditions in the neurology practice field, with very limited treatment options. FNDs present with sensory and/or motor symptoms that can mimic other neurological conditions, but appear to be related to recognizable psychological factors and are thought to occur via mechanisms other than those related to identifiable structural neuropathology. This condition has very limited treatment options, but there is preliminary evidence that psychedelic-assisted therapy (PAT) might be effective in a growing number of psychiatric disorders, including FNDs.

**Objectives:**

We aim to review the current literature regarding the role of psychedelic-assisted therapy in the treatment of functional neurological disorders.

**Methods:**

We search PubMed with the following keywords: psychedelics, functional neurological disorder and conversion disorder.

**Results:**

Only nine studies were published, between 1954 and 1967, reporting the use of psychedelics in the treatment of FNDs, with a total of 22 patients, of which 69% (*n* = 18) were found to have made at least some recovery, though the included studies were of low quality, often lacking control groups and valid outcome measures.

**Conclusions:**

There is a lack of evidence for the efficacy of PTA on the treatment of FNDs. Nevertheless, the discussion remains, as several abnormalities of the default mode network activity (DMN) have been reported in patients with FND and many of the proposed therapeutic benefits of psychedelics have been theorized to relate to their action on the DMN.

**Disclosure of Interest:**

None Declared

